# Cutaneous T-Cell Lymphoma and Cutaneous CAR-T Lymphoma in Patients with Other Malignancies

**DOI:** 10.3390/cancers18213527

**Published:** 2026-11-03

**Authors:** Caroline S. Myers, Elise A. Olsen, Christina Cruz, Ellen J. Kim, Tatyana Feldman, Alejandro Gru, Larisa J. Geskin

**Affiliations:** 1 Department of Dermatology, Perelman School of Medicine, University of Pennsylvania, Philadelphia, PA 19104, USA; christina.cruz3@pennmedicine.upenn.edu (C.C.); ellen.kim@pennmedicine.upenn.edu (E.J.K.); 2 Duke University Medical Center, Durham, NC 27710, USA; 3 Division of Lymphoma, John Theurer Center, Hackensack University Medical Center, Hackensack, NJ 07601, USA; tatyana.feldman@hmhn.org; 4 Department of Dermatology, Columbia University, New York, NY 10032, USA; aag2222@cumc.columbia.edu (A.G.); ljg2145@cumc.columbia.edu (L.J.G.)

**Keywords:** CTCL, lymphoma, CAR-T, malignancy, screening

## Abstract

Cutaneous T-cell lymphoma (CTCL) is a malignancy of skin-homing T cells associated with increased risk of second malignancies. CAR-T lymphoma is a mature T-cell malignancy arising in cancer patients treated with chimeric antigen receptor T-cell (CAR-T) therapy. Because of its frequent cutaneous involvement, CAR-T lymphoma can mimic CTCL, which in turn can resemble cutaneous sequelae of oncologic therapies. Careful evaluation of these entities in cancer patients is therefore essential, yet little guidance exists regarding optimal methods of screening, diagnosis, and treatment. Clinicians managing CTCL in cancer patients should consider how oncologic treatment toxicities may overlap with CTCL treatment toxicities or affect patients’ risk for other skin cancers. Where possible, immune-boosting or at least immune-preserving treatments are preferred, with immune checkpoint inhibition emerging as an important option in selected patients. Patients with a history of CAR-T therapy and new, persistent skin findings should undergo careful work-up with molecular screening for lymphoproliferative processes. Presence of a CAR in lesional tissue can be suggestive but is not diagnostic of a CAR-T-associated malignancy. Treatment for any patient with a cutaneous T-cell or CAR-T lymphoma and a second cancer should be an interdisciplinary effort tailored to the patient’s specific medical history and molecular profile. Further research is needed to establish better evidence-based guidelines for treatment of these patient populations.

## Simple Summary

Cutaneous T-cell lymphoma (CTCL) is an incurable cancer of skin-homing T cells that is associated with an increased risk of second malignancies. CAR-T lymphoma is a mature T-cell malignancy arising in cancer patients treated with chimeric antigen receptor T-cell (CAR-T) therapy. Few guidelines exist regarding management of CTCL or CAR-T lymphoma in cancer patients. This review uses the existing literature to provide a framework for the optimal evaluation, diagnosis, and treatment of these patients in the context of their complex clinical history and immune environment.

## 1. Introduction

Cutaneous T-cell lymphoma (CTCL) is a group of incurable cancers of skin-homing T cells that has been shown to confer an increased risk of other malignancies. In the Duke registry for patients with CTCL seen between 1966 and 1981 with median follow-up of 2.3 years, the development of a second cancer was noted to be 2.4 times higher than expected compared to the Connecticut tumor registry [1]. These findings are consistent with those of other groups [2,3] and emphasize the need for a clinical framework to guide CTCL management in cancer patients.

With the advent of Chimeric Antigen Receptor T-cell (CAR-T) therapy, another mature T-cell lymphoma has been observed in cancer patients: CAR-T lymphoma. Often presenting with dermatologic manifestations [4], CAR-T lymphoma can clinically and histopathologically mimic CTCL. As is the case for CTCL, few guidelines exist regarding how to diagnose and treat this rare mature T-cell lymphoma.

In this review, we attempt to provide a framework by which clinicians can consider how best to evaluate and manage CTCL and CAR-T lymphomas in patients with other malignancies. We begin by discussing available evidence guiding the management of CTCL in cancer patients, including recommendations for evaluation, screening, and treatment. We then discuss the management of CAR-T lymphoma in cancer patients, including clinical and laboratory evaluations as well as therapeutic options.

## 2. Oncogenesis of CAR-T Lymphomas, CTCL, and Second Malignancies

It is well-established that CTCL patients have an increased risk of developing a second malignancy. A 2021 meta-analysis reported a standardized incidence ratio (SIR) of 2.2 for all cancers, with 6–17% of patients developing a second malignancy and risk increasing with CTCL severity [3]. The risk is highest for hematologic malignancies, with Hodgkin and non-Hodgkin lymphomas occurring 15 times and five times more frequently than in the general population, respectively. Several solid tumors also occur more frequently in CTCL patients, including small-cell lung cancer (SIR 8.5), melanoma (SIR 3.1), and bladder cancer (SIR 1.7) [2].

The reason for this increased risk is unknown, but several factors could contribute. First, one must take into account the era in which data were accumulated, as the diagnostic parameters and treatments for CTCL have significantly changed over the past 50 years. Prior to 1974, lymph node or visceral involvement in CTCL was assumed to be an evolution to reticulum cell sarcoma, lymphosarcoma, lymphocytic lymphoma or Hodgkin’s disease [1]. The term CTCL was first introduced in 1975 by Edelson [5] primarily referring to its two most common subtypes, mycosis fungoides (MF) and Sezary Syndrome (SS). First in 1979 [6] and later in 2006 [7], there was a redefinition of CTCL clinical criteria that described extracutaneous disease in MF or SS, as well as other new subtypes of CTCL with diverse clinical presentation. Our understanding of what comprises a true second malignancy in CTCL has thus become more specific, excluding visceral manifestations of MF/SS or rare subtypes of CTCL that fall under a common umbrella of cutaneous lymphoproliferative disorders.

However, recent studies demonstrating the increased risk of second malignancy in CTCL patients utilize cohorts diagnosed by modern criteria [2,8], making misclassification alone an unlikely explanation for observed patterns. Alternatively, it is possible that CTCL treatments, through immunosuppression or cytotoxic activity, may increase the risk of a second malignancy. Prior to the introduction and off-label use of oral retinoids, interferon alfa, and photopheresis for MF/SS in the 1980s, systemic therapy for CTCL patients was primarily single- or multiagent chemotherapy. The first FDA-approved systemic medications for MF/SS were bexarotene and denileukin diftitox, both approved in 1999. The histone deacetylase inhibitors vorinostat and romidepsin were approved in 2006 and 2009, respectively, and mogamulizumab in 2018. Brentuximab was approved in 2017 for CD30+ CTCL. The NCCN now recognizes many other medications with off-label utility for various subtypes of CTCL [9]. All these medications have specific short- and long-term side effects, including the potential to induce other lymphoproliferative processes. However, there is no publication specifically addressing the risk of second malignancies using any of the agents FDA-approved in the past 20 years.

A third possibility is that immune dysregulation in CTCL patients [10,11] could create environments permissive to oncogenesis in other tissues. This theory is supported by the observation that the increased frequency of CTCL corresponds to age-related incidence of malignancy overall [12] possibly related to the age-related decrease in general immune surveillance [13]. Alternatively, molecular mechanisms driving CTCL oncogenesis could contribute to the development of second malignancies. The molecular landscape of CTCL is heterogeneous. Increased chromosomal instability results in frequent copy number variations in a variety of well-characterized oncogenes and tumor suppressors including *CDKN2A* and *TP53* [14]. Epigenetic studies of CTCL have demonstrated recurrent hypo- or hyper-methylation of cancer-related genes such as *PPARG* [15] or *FAS* [16] as well as up-regulation of oncogenic long noncoding RNAs such as MALAT1 [17]. Finally, telomerase activity has been shown to be up-regulated in CTCL [18], a characteristic promoting unrestrained cell proliferation in other cancers [19]. Any or a combination of these mechanisms could theoretically promote oncogenesis in CTCL and other malignancies. However, without studies characterizing the molecular overlap between CTCL and patient-matched second malignancies, it remains unclear how these molecular features might contribute to overall cancer risk.

The molecular features of CAR-T lymphomas in relationship to other cellular populations have been more well characterized. Although direct interruption of tumor suppressor loci by CAR-T integration is a putative driver of some CAR-T lymphomas [20], others demonstrate the presence of shared mutations in the CAR-T clone as well as non-malignant populations of lymphocytes [21] or CD34+ stem cells [22] present before CAR-T therapy. These reports suggest that, at least in a subset of patients, clonal hematopoiesis could contribute to the presence of a T-cell lymphoma in patients with a second malignancy.

Despite these theories, we wish to emphasize that the biology driving the increased incidence of second malignancies in patients with CTCL remains poorly understood. In this review, we therefore refer to malignancies co-occurring with CTCL as “second” rather than “secondary” malignancies, as we cannot infer causality from co-occurrence. Similarly, in our discussion of T-cell lymphomas arising after CAR-T therapy, we use the concise and common term “CAR-T Lymphoma” to refer to these clinical entities, although the directionality of the forces driving development of these diseases remains controversial. Excellent translational work is underway examining the drivers of T-cell lymphoma oncogenesis, but further research is required to elucidate the reasons for their co-occurrence with other malignancies.

## 3. Management of CTCL in Cancer Patients

CTCL is a chronic disease that often requires long-term management. As such, patients not only have a higher incidence of second malignancies, but also a longer duration of disease during which second malignancies might occur. This introduces unique challenges, as treatment decisions are complicated by overlapping toxicities, immune dysfunction, and the need to balance therapies directed at two separate diseases. Management therefore is often guided by general principles and clinical judgment on a case-by-case basis.

### 3.1. Screening and Surveillance

Despite the increased risk of other malignancies in CTCL, no formal surveillance guidelines exist. Current National Comprehensive Cancer Network (NCCN) recommendations only address lymphomatoid papulosis, which requires lifelong follow-up due to the risk of associated lymphoid malignancies [9]. Goyal et al. (2021) proposed a screening strategy that involves regular lymph node exams, skin checks, and smoking cessation counseling, given the increased risk of lymphomas, melanoma, and lung cancer, respectively [2]. The role of laboratory or imaging surveillance remains unclear and requires further investigation. All CTCL patients should be reminded about age-appropriate standard of care cancer screenings as per the United States Preventative Services Task Force, NCCN, and American Cancer Society guidelines for breast, colorectal, cervical cancer, lung and prostate cancer [23,24].

### 3.2. Principles of Managing CTCL with Concurrent Malignancy

No standard guidelines exist for the management of patients with CTCL and another malignancy, though general principles are commonly applied [25]. Treatment is generally directed first toward the more aggressive or life-threatening cancer. This assessment is often more art than science, especially in patients where both cancers are advanced. NCCN guidelines recommend multidisciplinary collaboration among dermatology, hematology/oncology, radiation oncology, and pathology, ideally with input from clinicians experienced in CTCL management [9]. Immune function plays a central role in this decision-making process. Since CTCL itself is associated with immune dysfunction, therapies that suppress immunity, such as chemotherapy, may worsen disease. When feasible, treatment strategies that preserve or boost immune function are favored [11].

One such option includes the use of immune checkpoint inhibitors (ICIs), which have emerged as an important tool in the treatment of multiple cancers [26]. There are currently 11 ICIs FDA-approved for 43 indications. Evidence has shown that PD-1/PDL1 inhibitors are safe and effective treatment options for at least a subset of CTCL patients, with more limited data supporting the use of CD47-SIRPα and CTLA4 blockade [27,28]. Perhaps the most well-studied ICI in CTCL is pembrolizumab. A phase 2 multicenter open-label trial (CITN-10) of pembrolizumab in 24 advanced-stage MF/SS patients with a median of 4 prior systemic therapies demonstrated a global overall response rate (ORR) of 38%, with two complete responses (CR) and seven partial responses (PR). The median duration of response (DOR) was not reached at median follow-up of 58 weeks [29]. A large real-world case series of 30 patients demonstrated an ORR of 48% (5 CR, 9 PR) with a median DOR of 13.1 months, median progression-free survival of 7.4 months, and median overall survival of 20.6 months [30]. Heavily pretreated patients or those with large cell transformation were less likely to respond. Another PD-1 antibody, tislelizumab, was studied in an open-label multicenter phase 2 trial in relapsed/refractory mature T- and NK-cell lymphomas of 77 patients. In Cohort 3 of MF/SS patients (N = 11), an ORR of 45.5% (1 CR), median DOR of 11.3 months, and median progression-free survival (PFS) of 16.8 months were observed with mostly grade 1 or 2 adverse events [31]. Nivolumab data in CTCL are more limited. The single prospective clinical trial with a dedicated MF cohort was a Phase Ib study in which 13 relapsed/refractory MF patients had an ORR of 15% [32]. One case report described a patient treated with nivolumab for their metastatic melanoma who also had concurrent Sezary syndrome that responded with treatment [33]. Finally, the PDL1 inhibitor durvalumab was combined with lenalidomide in a Phase 1 study of 12 evaluable patients with relapsed/refractory CTCL who saw an ORR of 53.8% and median DOR of 25.5 months [34]. In current NCCN guidelines for MF/SS CTCL subtypes, ICIs are listed as “other recommended” or “useful in certain circumstances” therapy options for Stage IB-IV disease. While risk of hyperprogression appears to be lower than anticipated for MF/SS, ICIs should be avoided in certain other CTCL subtypes (adult T-cell leukemia/lymphoma) [35].

### 3.3. Treatment Interactions and Overlapping Toxicities

Concurrent treatment of CTCL and a second malignancy introduces multiple challenges related to drug interactions and treatment-related toxicities. When choosing CTCL treatment regimens for patients with second malignancies, as well as while monitoring these patients for cancer therapy-related adverse cutaneous reactions, several factors should be considered.

First, phototherapy is a major component of CTCL management, but its use may be limited in patients with other cancers. PUVA is relatively contraindicated in patients with a history of melanoma or any active skin cancers because of its association with increased skin cancer risk [36]. Narrowband-UVB is also typically avoided in this population, though it can be used with caution in certain situations as it has not been definitively linked to increased skin cancer risk [37].

Systemic cancer therapies, including traditional chemotherapies as well as biologic agents, may cause cutaneous reactions that can impact clinical management of CTCL. In a single-center study of patients on chemotherapy, up to 40% developed various cutaneous reactions [38]. ICIs are associated with a wide spectrum of skin toxicities including pruritus, maculopapular rash, vitiligo, eczematous, lichenoid, urticarial, psoriasiform, and bullous dermatitis [39]. Mogamulizumab-associated rash occurs in approximately 20–60% of patients due to depletion of regulatory T cells [40]. All of these skin reactions can mimic CTCL, making assessment difficult. In the case of ICIs, cancer therapies may even exacerbate CTCL-related cutaneous inflammation. Fortunately, grade 1 and grade 2 skin reactions can often be managed conservatively to control symptoms enough to enable continuation of cancer therapies [41].

Overlapping toxicities are another concern when treating two cancers simultaneously. Many therapies used in CTCL and other cancers can cause photosensitivity, which limits phototherapy dosing and the co-administration of certain agents. Myelosuppression is another common adverse effect of CTCL therapies, including romidepsin, brentuximab vedotin, and interferon alfa [42,43,44]. Combining these drugs with chemotherapy may increase the risk of neutropenia and infections. Hepatotoxicity, metabolic disturbances, and peripheral neuropathy may similarly be amplified during concurrent treatment [45,46,47].

Radiation recall dermatitis is an inflammatory reaction triggered by systemic therapies at sites of prior radiation exposure. Because CTCL patients frequently receive localized radiation or total skin electron beam therapy, subsequent treatment with chemotherapy (most commonly including docetaxel, gemcitabine, doxorubicin, capecitabine, paclitaxel), targeted therapy (BRAF inhibitors), or immunotherapy (ICIs) for another malignancy may provoke this phenomenon [48,49,50].

Surgical management of a second malignancy may also require special consideration. Although surgery is generally well tolerated in early-stage disease, patients with extensive body surface area involvement or erythroderma have an increased risk of infection and sepsis. These patients may also have a higher risk of thrombosis and impaired wound healing [51]. CTCL-directed therapies may need to be withheld or modified in the perioperative period.

### 3.4. Implications for Clinical Trials and Transplantation

Concurrent malignancy has implications for eligibility for advanced therapies. Most oncology clinical trials restrict enrollment of patients with active second cancers, which may limit participation in both CTCL-specific trials and trials targeting the second malignancy [52,53].

A similar concern arises in patients eligible for hematopoietic stem cell transplant (HSCT), a common treatment for aggressive lymphomas. Although active malignancy is not explicitly named as a contraindication, functional status and life-limiting factors are heavily weighed for eligibility [53]. Additionally, the immunosuppression required for transplantation may allow progression of the second malignancy. Mogamulizumab and immune checkpoint inhibitors administered soon before allogeneic HSCT significantly increase the risk of GVHD and mortality [54,55].

## 4. Management of CAR-T Lymphomas in Cancer Patients

Chimeric antigen receptor T-cell (CAR-T) therapy is a powerful treatment for multiple liquid malignancies with burgeoning applications in autoimmunity [56] and solid tumors [57], as well. However, rare cases of CAR+ T-cell lymphoma arising after CAR-T therapy have been reported in the literature, generating substantial clinical, scientific, and regulatory concern. Cutaneous involvement of these CAR-T lymphomas is prominent among published cases and may provide early, accessible evidence of disease. Therefore, cutaneous CAR-T lymphoma has become an increasingly important entity for dermatologists, oncologists and other clinicians to recognize, diagnose and treat.

### 4.1. Dermatologic Manifestations and the Role of Skin-Directed Evaluation

Skin involvement is disproportionately prominent in the small number of molecularly characterized CAR-T lymphomas. In a published compilation of ten CAR-positive cases, Braun et al. identified cutaneous involvement in four, either as isolated primary cutaneous disease or as part of nodal, leukemic, or gastrointestinal lymphoma [4]. Skin lesions were also present in all three cilta-cel cases described by two groups [21,58] and in the sole case occurring amongst patients in a French CAR-T patient registry [59]. A dedicated dermatology commentary has consequently called attention to the cutaneous presentation of these malignancies [60].

The reported cutaneous morphologies of CAR-T lymphoma are diverse and include erythematous or violaceous plaques, papules, nodules, tumors, and subcutaneous lesions. Facial and head-and-neck involvement has appeared in several reports and in emerging institutional cohorts [21,58], but the published denominator is too small to conclude that there is a true facial predilection. These patterns should be described as emerging observations rather than defining diagnostic criteria.

Dermatologists have an important role because skin may provide the first visible evidence of disease and the most accessible tissue for repeated sampling. A new persistent plaque, nodule, tumor, or unexplained eruption arising months or years after CAR-T should not automatically be attributed to drug eruption, infection, immune reconstitution, leukemia cutis, recurrence of the original malignancy, or nonspecific dermatitis. Biopsy is warranted when lesions are persistent, progressive, infiltrated, nodular, ulcerated, treatment-resistant, or accompanied by lymphocytosis, gastrointestinal symptoms, adenopathy, cytopenias, or constitutional changes (Box 1).

Box 1 **Evaluation of a suspected post-CAR-T T-cell neoplasm.**

1. Confirm persistent or progressive clinical disease and obtain adequate tissue.
2. Perform expert pathology review, broad immunophenotyping, TCR clonality, and EBV testing when appropriate.
3. Estimate tumor purity and preserve fresh, frozen, and formalin-fixed material whenever possible.
4. Test tumor-enriched material for CAR DNA, RNA, or protein using at least two complementary methods.
5. Compare the malignant TCR clone across tumor, blood, marrow, CSF, pre-apheresis material, and final CAR-T product when available.
6. Perform integration-site mapping and tumor genomic profiling in cases with convincing CAR involvement.
7. Classify lymphoma diagnosis, product derivation, and inferred mechanism separately.
8. Report the event to the manufacturer and appropriate pharmacovigilance authorities.

At the same time, cutaneous lymphoma must not be over-diagnosed. CAR-T therapy is associated with a broader spectrum of dermatologic adverse events, including inflammatory eruptions, severe cutaneous reactions, vascular manifestations, infections, and treatment-related toxicities [61]. Therapeutic CAR-T cells can traffic into inflamed skin and produce CAR positivity in a biopsy without being malignant. Clinicopathologic correlation and longitudinal observation are therefore especially important in skin-limited disease.

A dermatologic evaluation should document lesion morphology, distribution, time of onset relative to CAR-T and subsequent immune therapies, associated symptoms, treatment response, and evidence of extracutaneous disease. Whenever possible, adequate tissue should be preserved for routine histology, immunohistochemistry, TCR sequencing, targeted DNA and RNA studies, and CAR testing. If the initial biopsy is small or heavily inflamed, a repeat biopsy from a more developed, untreated lesion may be more informative. Fresh tissue for flow cytometry or cryopreservation should be obtained when feasible, particularly if integration-site or single-cell studies are anticipated.

### 4.2. Diagnostic Strategy and Levels of Evidence

A rigorous diagnostic evaluation should distinguish assays that detect the presence of CAR sequences from assays that link those sequences to malignant cells and assays that address causality (Table 1).

Histopathology remains the starting point. Tissue should be reviewed by a hematopathologist or dermatopathologist familiar with both T-cell lymphoma and post-immunotherapy changes. The immunophenotypic panel should be adapted to the site and morphology but generally includes CD2, CD3, CD4, CD5, CD7, CD8, CD30, TCR-beta F1, TCR-delta, cytotoxic markers, Ki-67, and appropriate markers to rule out Epstein–Barr or other viral causes. Because many patients undergoing CAR-T therapy are receiving treatment for a B-cell malignancy, cutaneous manifestation of their primary malignancy should also be ruled out.

For patients with blood involvement, flow cytometry can identify an aberrant T-cell population and permit sorting for downstream analysis. T-cell receptor (TCR) sequencing establishes clonality and permits comparison among skin, lymph node, blood, marrow, gastrointestinal tissue, cerebrospinal fluid, apheresis material, and CAR-T product. Whole-genome or targeted DNA sequencing can be used to assess somatic mutations, structural variants and copy number changes. It is important to note that matched germline or non-tumor material is essential for distinguishing somatic tumor variants from inherited changes. Peripheral blood may not serve as a clean germline comparator when clonal hematopoiesis is present; in these cases, buccal samples, cultured fibroblasts, or another nonhematopoietic source may be needed. Additionally, many oncogenic mutations associated with CAR-T lymphoma are also drivers of other lymphoproliferative disorders [62]; thus, mutational profiling can be a helpful tool but does not distinguish sporadic from product-derived lymphomas.

Several assays can be used to test for the presence of a CAR. However, they do not specifically link CAR expression to malignant cells, and thus should be interpreted relative to tumor purity and ideally performed on microdissected or sorted malignant T cells. CAR detection by quantitative PCR (qPCR) or droplet digital PCR (ddPCR) is highly sensitive [63]. Targeted RNA sequencing may identify CAR transcripts or host–vector chimeric reads. Flow cytometry is perhaps the most accessible way to directly associate CAR protein with an aberrant immunophenotype when validated reagents are available [64].

Although difficult and expensive to incorporate into routine clinical practice, several techniques utilized in translational research remain the gold standard for diagnosis and characterization of CAR-T lymphomas. Single-cell RNA/TCR analysis can link CAR expression, clonotype, phenotype, and transcriptional state in the same cells and therefore provide powerful evidence of product derivation [21]. Integration-site analysis remains the strongest method for defining product origin. Techniques include linear amplification-mediated PCR, nonrestrictive LAM-PCR, linker-mediated PCR, anchored capture, targeted vector-junction sequencing, whole-genome sequencing, and long-read sequencing [65,66,67]. Each method has biases, particularly when DNA is fragmented or when the exact vector reference is unavailable. Negative testing is interpretable only when tumor fraction, nucleic-acid quality, assay coverage, and positive controls are adequate.

The ideal analysis compares three time points: material before or at apheresis, the final CAR-T product, and the post-treatment tumor. No published case has yet received comprehensive uniform analysis at all three stages [4]. Residual commercial product is often inaccessible because of contractual, regulatory, or manufacturer restrictions. Establishing mechanisms for rapid manufacturer collaboration and product-remnant access is therefore a major research priority.

### 4.3. Differential Diagnosis

The differential diagnosis depends on the tissue and presentation. Clinical or histopathologic mimics without CAR expression include reactive immune proliferations, drug eruptions, viral infections, Epstein–Barr virus-associated lymphoproliferation, conventional sporadic T-cell lymphoma, recurrent original malignancy, therapy-related myeloid neoplasia with secondary lymphoid abnormalities, and inflammatory toxicities involving skin or gastrointestinal tract. Benign mimics with CAR expression can include physiologic or exaggerated persistence of CAR-positive therapeutic clones [68], including oligoclonal or monoclonal CAR-T expansions, as well as reactive clonal expansions of non-CAR-T cells triggered by infection, inflammation, or immune reconstitution. Thus, clonality alone does not establish lymphoma, and CAR positivity alone does not establish malignant transformation.

WHO and International Consensus Classification categories should be applied where appropriate [69], but the existing schemes were not designed around engineered-cell origin. A tumor population may morphologically and/or immunophenotypically resemble PTCL, T-LGLL, indolent gastrointestinal T-cell lymphoma, primary cutaneous anaplastic large-cell lymphoma, or a T-follicular helper-lineage malignancy while also carrying the etiologic descriptor “CAR-T derived.” Morphologic classification and origin assignment should therefore be reported as complementary layers rather than competing diagnoses.

### 4.4. Treatment and Clinical Course

No standard treatment exists because reported cases differ biologically and clinically. Aggressive lymphomas have been treated with multiagent chemotherapy, regimens containing the anti-CD2 monoclonal antibody alemtuzumab, radiation, extracorporeal photopheresis, interferon, targeted agents, and allogeneic transplantation. Indolent gastrointestinal or cutaneous cases have responded variably to corticosteroids, calcineurin inhibitors, methotrexate, the anti-CD30 monoclonal antibody conjugate brentuximab vedotin, and other immunomodulatory approaches [4,20,21,22,58,59,70].

CD30 expression offers a rational therapeutic target in select cutaneous or systemic cases. CCR4 expression may support the use of mogamulizumab, while JAK/STAT alterations may create opportunities for pathway inhibition. Treatment should be based on clinical behavior, compartment, phenotype, genomic vulnerabilities, status of the original malignancy, and the patient’s immune and marrow reserve.

## 5. Conclusions

Management of CTCL or CAR-T lymphoma in a patient with concurrent malignancy is complex and supported by limited evidence. The forces driving the co-occurrence of these T-cell lymphomas with other solid or liquid malignancies remain unclear, although new molecular studies provide some insight. Because these T-cell lymphomas can resemble each other as well as cutaneous sequelae of oncologic treatment, careful diagnostic evaluation of patients with new cutaneous symptoms is essential. Management of CTCL in cancer patients hinges on careful consideration of overlapping treatment toxicities. Evidence guiding CAR-T lymphoma management is more sparse, but many treatments discussed in the literature overlap with those used for CTCL. Treatment for these complex patients often requires consideration on a case-by-case basis, but general principles are summarized in Table 2. Most importantly, management of patients with CTCL and/or cutaneous CAR-T lymphoma in the context of second malignancies relies on patient-specific decision-making and collaboration between specialties. Additional research is needed to inform clinical guidelines for this growing patient population.

## Figures and Tables

**Table 1:** Diagnostic assays and their evidentiary value.

Principal Question	Method	Strength	Key Limitation
Is the process morphologically and phenotypically malignant?	Histopathology and immunohistochemistry	Defines lymphoma type and tumor content	Indolent and reactive processes may overlap
Is there an aberrant T-cell population?	Flow cytometry	Links phenotype to a discrete population	Limited by tissue viability and available CAR reagents
Is the population clonal? Is the same clone present across samples?	TCR sequencing	Essential for lineage tracking	Clonality alone does not prove malignancy
Is the CAR sequence present in the specimen?	Bulk CAR qPCR/ddPCR	Sensitive and feasible on limited material	Cannot distinguish malignant from admixed CAR-T cells
Is the CAR expressed?	Targeted RNA sequencing	Can identify expression and chimeric reads	Bulk signal may arise from bystander cells
Where did the CAR vector integrate in the genome?	Integration-site mapping	Strongest conventional evidence of derivation	Technically biased; negative results may be noninformative
Is the CAR present in tumor-enriched cells?	Microdissected or sorted-cell CAR testing	Strong evidence of product origin	Tumor purity may vary
Is the CAR present in clonal malignant cells?	Single-cell RNA/TCR/CAR profiling	Direct clone-level linkage	Expensive and requires viable/high-quality material
Where are abnormal CAR-positive cells located in tissue?	Spatial transcriptomics	Preserves tissue context	Expensive; does not necessarily map genomic integration
What are the integrations, structural changes, and clonal architecture?	WGS or long-read sequencing	Broad mechanistic assessment	Material-intensive and analytically complex

**Table 2:** Recommendations for approaches to different clinical scenarios.

Scenario	Recommendation
New cutaneous symptoms in a cancer patient after CAR-T therapy	Careful clinical evaluation with potential biopsy for histology and immunohistochemistry. TCR sequencing if there is suspicion for a lymphoproliferative process, with subsequent molecular evaluation for the presence of a CAR if clonality is confirmed.
New cutaneous symptoms in a cancer patient with a history of CTCL	Careful clinical evaluation with potential biopsy for histology, immunohistochemistry and TCR sequencing to distinguish CTCL flares from cutaneous sequelae of cancer treatment.
New cancer diagnosis in a CTCL patient	Prioritization of treatment for the more aggressive cancer, with careful consideration of overlapping treatment toxicities.
Cancer patient with CTCL requiring skin-directed therapy	Avoidance of UV therapy in patients with UV-induced cancers (e.g., melanoma, non-melanoma skin cancer) or in patients receiving oncologic therapies increasing their UV susceptibility. Multidisciplinary discussion about timing of skin radiation therapy in patients receiving oncologic therapies increasing their risk of radiation-related toxicity.
Cancer patient with CTCL requiring systemic therapy	Opting for therapies that can treat both cancer types where possible. Consider immune-preserving or immune-boosting therapies, such as immune checkpoint inhibition, where applicable.

## Data Availability

No new data were created or analyzed in this study. Data sharing is not applicable to this article.

## Abbreviations

The following abbreviations are used in this manuscript:

CTCL	Cutaneous T-cell Lymphoma
CAR-T	Chimeric Antigen Receptor T cell
MF	Mycosis Fungoides
SS	Sezary Syndrome
NCCN	National Comprehensive Cancer Network
SIR	Standardized Incidence Ratio
CR	Complete Response
PR	Partial Response
DOR	Duration of Response
PFS	Progression-Free Survival
ORR	Overall Response Rate
PUVA	Psoralen plus Ultraviolet-A Radiation
ICI	Immune Checkpoint Inhibitor
HSCT	Hematopoietic Stem Cell Transplant
TCR	T-cell Receptor
ddPCR	Droplet Digital Polymerase Chain Reaction
qPCR	Quantitate Polymerase Chain Reaction
